# Recombinant activated factor VII in cardiac surgery – first, do no harm

**DOI:** 10.1186/1749-8090-2-50

**Published:** 2007-11-24

**Authors:** Oliver J Warren, Ara W Darzi, Thanos Athanasiou

**Affiliations:** 1 Department of BioSurgery and Surgical Technology, Imperial College London, 10^th ^Floor QEQM Wing, St. Mary's Hospital, Praed Street, London, W2 1NY, UK

## Letter to the editor

We read with interest Heise et al's case report and review of the literature regarding the use of recombinant activated factor VII (rFVIIa) in patients with ventricular assist devices (VAD) [1]. Whilst we welcome the addition to the literature in this area, there are two key areas regarding this relatively novel haemostatic agent that we felt the authors had failed to address appropriately, and that may therefore mislead the practicing cardiac surgeon.

## RFVIIa's method of action; the source and role of tissue factor in cardiac surgery patients

In their discussion, the authors state that *'the effect of rFVIIa on plasmatic coagulation derives from its interaction with tissue factor. Thus, the pro-coagulatory effect is predominantly located in regions where tissues or vessels are injured'*. This is a simplification of a highly complex and only partially understood process. The authors have based their statement on what we know of endogenous Factor VIIa and Tissue Factor (TF) interaction in physiological conditions, where TF is present as an inactive pool on sub-endothelial cells. Vessel injury exposes this TF to the blood, where it binds and activates FVII. The resulting TF-FVIIa complex catalyzes the conversion of factor X into its active form (Xa), leading to thrombin formation and platelet activation. This creates a surface that supports the binding of coagulation factors and thereby facilitates the full thrombin burst necessary for haemostasis.

One cannot presume that the pro-coagulatory effect of rFVIIa occurs in the same way in patients undergoing VAD surgery; significant controversy exists surrounding both the source and the role of tissue factor in this setting. The systemic inflammatory response witnessed in patients undergoing major cardiac surgery involving artificial circulatory support has a profound impact on the coagulation system and thus TF expression is highly unlikely to be restricted to the sub-endothelium. Several groups of investigators have reported the presence of physiologically active 'blood-borne TF' in pro-inflammatory conditions, including cardiac surgery [2-4]. What form this takes remains unclear; blood-borne TF has been reported as being located on blood cells, being an undefined mixture of pro-coagulant micro-particles (0.1 to 1 μm) or being soluble pro-coagulant TF fragments [5-7]. Pro-inflammatory cytokines can stimulate neutrophils and monocytes to produce and present TF on their surface [8,9] and blood-borne TF in combination with activated monocytes may activate FVII in cardiac surgical patients more than when combined with activated platelets [2,10]. Furthermore, many patients undergoing VAD surgery suffer from ischaemic cardiomyopathy. Within atherosclerotic plaques, vascular smooth muscle cells, monocytes and endothelial cells have all been reported to aberrantly express and expose TF to the circulation [11]. Not only has this been shown to be a critical event in atherothrombosis, but this expression and exposition has been shown to occur at higher levels in patients with symptomatic coronary disease, suggesting a role for TF in plaque instability [12].

The exact role of TF in rFVIIa's effect also requires further elucidation. The high plasma concentrations of rFVIIa required to induce haemostasis in refractory haemorrhage suggests that TF-dependent activation of the coagulation cascade cannot be the sole mechanism of action. It has been shown that rFVIIa is able to directly activate Factor X on phospholipid vesicles, activated platelets and monocytes, independently of TF [13-15], although TF-independent generation of thrombin is much less efficient. Whilst the authors alluded to TF-independent thrombin generation in their introduction we feel this point must be emphasised.

## Patient safety – the risk of thromboembolic adverse events

We believe the authors have significantly underestimated both the frequency and seriousness of the risk of thromboembolic (TE) complications in cardiac surgery patients. They refer to *'the relatively low incidence of thromboembolic events (1–2%) after the use of rFVIIa' *in Levi et al's systematic review of the literature [16], but neglect to inform the reader that over 50% of the data included in this synthesis came from case reports or series. Furthermore less than 5% of the patients in this article were surgical patients, the vast majority being haemophiliacs, in which rFVIIa is a licensed treatment for haemorrhage. These are both crucial factors, as they introduce a high risk of both publication and selection bias. Heise et al infer that because TE complications in patients with VAD were not explicitly mentioned in O'Connell et al's paper in 2006 [17] they 'seem to be very rare'. This conclusion simply cannot be drawn from the available data. Whilst the authors refer to this paper a second time, they fail to emphasise that O'Connell et al stressed that most TE adverse events follow the use of rFVIIa for unlabeled indications, and result in serious morbidity and mortality.

It is difficult to know what the real risk of serious TE adverse events in this cohort of patients is. In a systematic data synthesis of the cardiac surgery literature performed early this year, our group reported a TE adverse event rate in adult patients treated for refractory haemorrhage of 5.3% [18]. This was very similar to the 6% quoted by Levy et al who reviewed the critical safety data from 13 rFVIIa clinical trials in patients with coagulopathy secondary to anti-coagulation, cirrhosis, or severe traumatic injury [19]. However, we would stress that even these studies are likely to suffer from underreporting. Whatever the rate, the morbidity and mortality of spontaneous arterial or venous thrombosis are severe, and must not be understated.

## Conclusion

It is likely that the level and location of TF expression in patients undergoing VAD surgery is significantly higher and more widespread than in normal subjects, and thus these patients are at risk of spontaneous intra-vascular thrombosis if administered rFVIIa. The level of this risk is currently unquantifiable. We agree wholeheartedly with the authors' conclusion that rFVIIa is a promising therapeutic option when conventional treatments for refractory haemorrhage have been exhausted, and use it in our own clinical practice. However we wish to emphasise that careful risk-benefit analysis must be made before prescribing rFVIIa to any bleeding cardiac patient. Evidence-based recommendations regarding the use of rFVIIa in cardiac surgery are available to the practicing cardiac surgeon or anaesthetist and we refer our colleagues to these guidelines [20-22].

## Competing interests

OW and TA have both received an unrestricted educational grant from NovoNordisk^®^, the manufacturers of Novoseven, to fund their research in this area. The company has had no input into this letter.

## Authors' contributions

OW and TA were equally involved in the conceptualization, research, writing and manuscript preparation of this letter. AD was responsible for manuscript revision and important intellectual contact. TA is the guarantor. All authors read and approved the final manuscript.
